# Lysosomal acid phosphatase 2 is an unfavorable prognostic factor but is associated with better survival in stage II colorectal cancer patients receiving chemotherapy

**DOI:** 10.18632/oncotarget.14552

**Published:** 2017-01-06

**Authors:** Yu-Chieh Lee, Chia-Yu Su, Yuan-Feng Lin, Chun-Mao Lin, Chih-Yeu Fang, Yen-Kuang Lin, Michael Hsiao, Chi-Long Chen

**Affiliations:** ^1^ Graduate Institute of Medical Sciences, College of Medicine, Taipei Medical University, Taipei, Taiwan; ^2^ Genomics Research Center, Academia Sinica, Taipei, Taiwan; ^3^ Graduate Institute of Clinical Medicine, Taipei Medical University, Taipei, Taiwan; ^4^ Department of Biochemistry, School of Medicine, Taipei Medical University, Taipei, Taiwan; ^5^ Department of Pathology, College of Medicine, Taipei Medical University, Taipei, Taiwan; ^6^ Department of Pathology, Taipei Medical University Hospital, Taipei, Taiwan; ^7^ Department of Pathology, Wan Fang Hospital, Taipei Medical University, Taipei, Taiwan; ^8^ Biostatistics Center, Taipei Medical University, Taipei, Taiwan; ^9^ Division of Gastroenterology, Department of Internal Medicine, Shuang Ho Hospital, Taipei Medical University, Taipei, Taiwan; ^10^ Department of Biochemistry, College of Medicine, Kaohsiung Medical University, Kaohsiung, Taiwan

**Keywords:** colorectal carcinoma, lysosomal acid phosphatase 2, 5-FU, chemotherapy

## Abstract

Colorectal cancer (CRC) is one of the leading cancers worldwide. Surgery is the main therapeutic modality for stage II CRC. However, the implementation of adjuvant chemotherapy remains controversial and is not universally applied so far. In this study, we found that the protein expression of lysosomal acid phosphatase 2 (ACP2) was increased in CRC and that stage II CRC patients with high ACP2 expression showed a poorer outcome than those with low ACP2 expression (*p* = 0.004). To investigate this discrepancy, we analyzed the relation between ACP2 expression and several clinical cofactors.

Among patients who received chemotherapy, those with an high expression of ACP2 showed better survival in both stage II and III CRC than those with low ACP2 expression. In stage II CRC patients, univariate analysis showed ACP2 expression and T stage to be cofactors significantly associated with overall survival (ACP2: *p* = 0.006; T stage: *p* = 0.034). Multivariate Cox proportion hazard model analysis also revealed ACP2 to be an independent prognostic factor for overall survival (ACP2: *p* = 0.006; T stage: *p* = 0.041). Furthermore, ACP2-knockdown CRC cells showed an increase in chemoresistance to 5-FU treatment and increased proliferation marker in the ACP2 knockdown clone.

Taken together, our results suggested that ACP2 is an unfavorable prognostic factor for stage II CRC and may serve as a potential chemotherapy-sensitive marker to help identify a subset of stage II and III CRC patients for whom chemotherapy would improve survival.

**Highlights:**

1. To the best of our knowledge, the study is the first report to show ACP2 overexpression in human colorectal cancer (CRC) and its association with poor outcome in stage II CRC.

2. Patients with stage II and III CRCs with high expression of ACP2 were more sensitive to chemotherapy than those with a low expression.

3. ACP2 expression may serve as a marker for CRC patients receiving chemotherapy and help identify the subset of CRC patients who would benefit from chemotherapy.

## INTRODUCTION

Colorectal cancer (CRC) is the third common cancer the second leading cause of cancer-related death in the Western world, with more than 600,000 deaths worldwide each year [1]. In Taiwan, CRC is the second most common cancer after breast cancer and the third most common cause of cancer mortality after carcinomas of the lung and liver. Death resulting from CRC is associated with the disease stage, a more advanced grade, and the presence of obstruction [2]. Adjuvant chemotherapy after surgical resection has been demonstrated to improve survival in stage III colon cancer.

Early stage CRC is amenable to surgery and chemotherapy. These acts in a complementary way can reduce the risk of local recurrences or distal metastasis. The current standard protocol of care for patients with high-risk stage II and stage III CRC is 5-fluorouracil (5-FU) or 5-FU/oxaliplatin chemotherapy following surgery [3]. A number of other cancer treatments, such as breast, esophageal, and gastric cancers, also generally include 5-FU in combination with other drugs [4]. However, even with chemotherapy, treatment for CRC may still result in failure because of the resistance of the tumor. Indeed, low response rates of 20%–30% have been reported in metastatic CRC patients [5]. Even with the improvement of therapeutic modality in recent years, including the development of new chemotherapeutic agents, patient mortality from CRC remains very high. It is, therefore, vital to have reliable biomarkers to identify the subsets of CRCs that show a good response to chemotherapy; such biomarkers will eventually bring important benefits for patients. A number of studies have been undertaken to clarify the therapeutic and molecular determinants of individual failures in response to 5-FU-based chemotherapy in CRC, but as yet this aim has not been sufficiently achieved for the development of targeted therapy and personalized medicine [6].

Lysosomal acid phosphatase 2 (ACP2), composed of alpha and beta subunits, is a lysosomal enzyme that usually serves as a biochemical marker for this organelle. The ACP2 gene encodes the major beta subunit of lysosomal acid phosphatase in humans. ACP2 is associated with several defects including bone structure alterations, lysosomal storage defects [7], and abnormal development of the central nervous system [8]. Deletion of ACP2 resulted in cerebellum abnormalities, delayed growth, hair disease, metabolic disorder [9], and an ataxia-like phenotype in *nax* mice [10]. Using cDNA microarray analysis, an increased ACP2 expression has been reported in oral squamous cell carcinoma [11]. The study of ACP2 expression in human basal cell carcinoma and normal skin tissue by cDNA microarray also showed similar results. These results indicated a high expression level of ACP2 may occur in human malignant tumors. The exact physiological and biochemical functions of ACP2 remain unclear. The roles of ACP2 in human malignancy are also undecipherable.

In the present study, we showed ACP2 to be an unfavorable prognostic factor for patients with stage II CRC. More importantly, high expression of ACP2 served as chemosensitive marker in both stage II and III CRC; patients with high ACP2 expression achieved better survival than those with low ACP2 expression. These findings were partly supported by the finding that an *ACP2*-knockdown CRC cell clone showed increased chemoresistance to 5-FU treatment. Hence, ACP2 may serve as a potential chemosensitive marker to identify subsets of CRC patients who would benefit from appropriate chemotherapy.

## RESULTS

### Correlation between ACP2 expression and prognosis in stage II and III CRC patients

**Figure 1:**
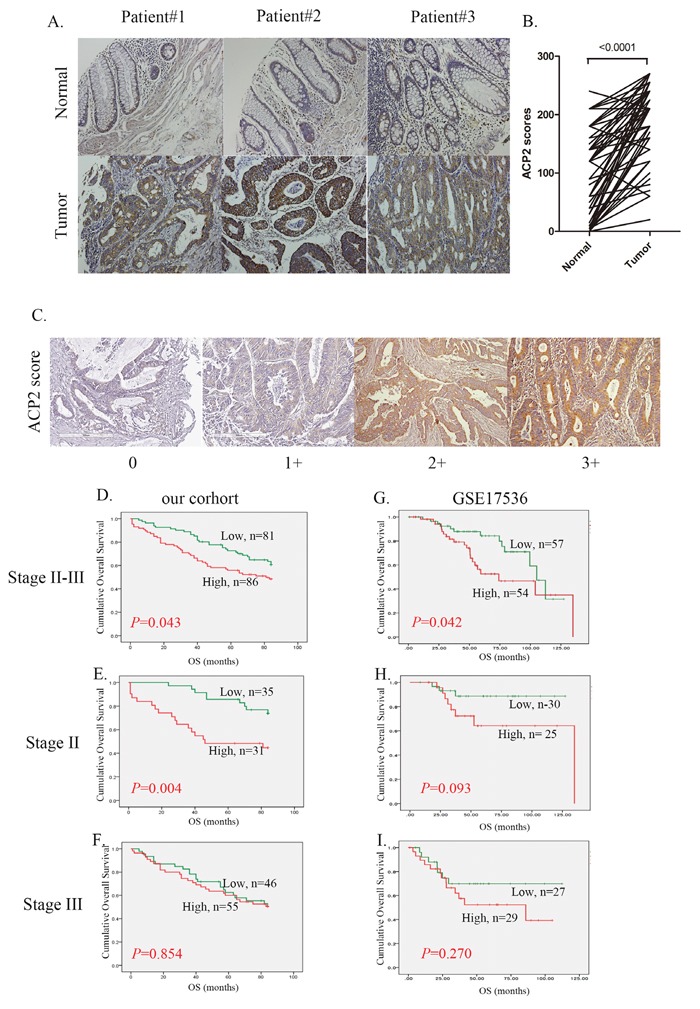
Correlation between ACP2 expression and prognosis in stage II and III CRC patients **A**. Representative images from immunohistochemical (IHC) staining of ACP2 proteins in matched primary colon tumors and normal adjacent tissues. **B**. Quantification of cytoplasmic IHC expression of ACP2 in primary colorectal tumors in comparison with paired normal tissues. The scores were calculated as staining intensity multiplied by the percentage of stained cells. **C**. Scores indicating ACP2 levels in representative colorectal tumor tissues. **D, E**, and **F**. Kaplan–Meier plots of overall survival for stage II and III colorectal carcinoma combined, stage II alone, and stage III alone, with the colorectal carcinoma stratified by ACP2 level for the cohort of the present study. **G, H**, and **I**. The equivalentKaplan–Meier plots for the GSE17536 dataset. The differences between groups were tested using log rank tests.

The relevant clinicopathological data of the 167 CRC patients whose specimens were included in this study are summarized in Supplementary Table 1. To determine the prognostic significance of ACP2 expression in CRC, the tissues were examined by IHC staining. We first examined 76 sets of matched samples from primary CRC tumors and non-tumor colon tissues to be sure of the expression pattern of ACP2. ACP2 expression was found increased significantly in tumors as compared with non-tumor colon tissues (Figure 1A and 1B, p < 0.0001). Figure 1C showed how ACP2 staining intensity was defined from levels 0 to 3+.

We next investigated the relationship between ACP2 expression levels and the survival of CRC patients. Overall, 86 patients with high expression level of ACP2 (scores of 2+ or 3+) had poor overall survival (OS) as compared with the other 81 patients with low expression level of ACP2 (with scores of 0 or 1+) (Figure 1D). In addition, we analyzed the association between ACP2 expression and patients’ survival at different disease stages. There was a significant association between ACP2 expression and poor overall survival in stage II CRC patients (*P* = 0.004; Figure 1E), but not in stage III patients (*P* = 0.854; Figure 1F). To confirm the prognostic value of ACP2, we performed additional survival analysis using the GEO dataset GSE 17536 [12]. This RNA array dataset consists of 111 cases of stage II and III CRC. Among them, 54 patients with high expression levels of ACP2 (greater than the median value) had poor outcome as compared with the other 57 patients with low ACP2 expression (lower than the median value) (*P* = 0.042, Figure 1G). Similarly, the overall survival of stage II CRC patients with high ACP2 expression had a tendency towards poor prognosis (*P* = 0.093, Figure 1H), but again this was not the case with stage III CRC patients (*P* = 0.281, Figure 1I). These results were consistent with our clinical observations in a seven-year retrospective record (Figure 1D, 1E, 1F and 1G, 1H, 1I). We therefore concluded that high expression of ACP2 is an unfavorable prognostic marker for CRC, specifically in stage II CRC patients. The univariate and multivariate Cox proportional hazards analyses further illustrated the association of ACP2 expression with cancer mortality (Table 1). In the univariate analysis, high expression of ACP2 (HR, 1.597; 95% confidence interval (CI), 1.008–2.530; *P* = 0.046) was significantly associated with survival. In the multivariate model, chemotherapy was an independent prognostic classifier (HR, 0.479; 95% CI, 0.273–0.841; *P* = 0.010). Although the association with ACP2 expression did not reach statistical significance, there was a tendency that could indicate it to be a prognostic classifier (*P* = 0.052). However, ACP2 expression was associated with survival in stages II and III of CRC patients (Figure 1D). From these data, high expression of ACP2 correlates significantly with poor outcome and is an independent prognostic factor in stage II CRC.

**Table 1:** Univariate and multivariate analyses of ACP2 with regard to OS in stage II and III colorectal cancer

Variables	Comparison	HR^a^	95% CI^b^	*P*	HR^a^	95% CI^b^	*P*
Cox Univariate analysis (OS)				Cox Multivariate analysis (OS)			
Stage (AJCC)	II vs. III	1.251	0.781–2.005	0.352	0.307	0.040–2.364	0.257
Tumor	T12 vs. T34	1.560	0.870–2.796	0.136	0.791	0.304–2.059	0.631
Node	Yes; No	1.303	0.809–2.096	0.276	4.831	0.592–39.410	0.141
Emboli	Yes; No	1.226	0.768–1.957	0.394	1.258	0.748–2.115	0.386
Perineural	Yes; No	1.370	0.827–2.268	0.221	1.425	0.828–2.452	0.201
Chemotherapy	Yes; No	0.745	0.470–1.179	0.209	0.479	0.273–0.841	0.010
ACP2	Low; High	1.597	1.008–2.530	0.046	1.601	1.000–3.213	0.052

^a^ Hazard ratio (HR) estimated from the Cox proportional hazard regression model.

^b^Confidence interval of the estimated HR.

ACP2: Acid phosphatase 2; OS: overall survival

### Correlation between ACP2 expression and survival in stage II and III CRC patients

To clarify the prognostic role of ACP2 in patients with different stage of CRC, we examined the independent prognostic value of ACP2 by Cox regression analysis separately in stage II and stage III CRC patients. In the univariate analysis, high expression of ACP2 (HR: 3.111; 95% CI: 1.382–7.005; *P* = 0.006; Table 2) and T stage (HR: 3.705; 95% CI: 1.101–12.462; *P* = 0.034) were significantly correlated with OS in patients with stage II CRC. In the multivariate analysis, OS was significantly associated with ACP2 expression (HR: 2.885; 95% CI: 0.799–10.419; *P*= 0.006) and T stage (HR: 3.742; 95% CI: 1.058–13.233; *P* = 0.041). However, in stage III CRC patients, ACP2 was not significant in either the univariant or the multivariant analysis. Notably, chemotherapy was the only valid prognostic factor in stage III patients with CRC (HR: 0.456; 95% CI: 0.227–0.917; *P* = 0.027, Table 3). These results suggested that ACP2 expression and T stage were prognostic classifiers in stage II CRC patients. Conversely, chemotherapy, which was not commonly used in stage II CRC, appeared to be a prognostic factor for stage III patients of CRC. These seemingly paradoxical results deserved further investigation.

**Table 2:** Univariate and multivariate analyses of ACP2 with regard to OS in stage II colorectal cancer

Variables	Comparison	HR^a^	95% CI^b^	*P*	HR^a^	95% CI^b^	*P*
Cox Univariate analysis (OS)				Cox Multivariate analysis (OS)			
Tumor	T3 vs. T4	3.705	1.101–12.462	0.034	3.742	1.058–13.233	0.041
Emboli	Yes; No	0.778	0.347–1.747	0.543	0.798	0.314–2.031	0.636
Perineural	Yes; No	0.689	0.163–2.916	0.612	0.882	0.203–3.819	0.866
Chemotherapy	Yes; No	0.557	0.223–1.389	0.210	0.566	0.204–1.574	0.276
ACP2	Low; High	3.111	1.382–7.005	0.006	3.274	1.413–7.586	0.006

^a^ Hazard ratio (HR) estimated from Cox proportional hazard regression model.

^b^ Confidence interval of the estimated HR.

ACP2: Acid phosphatase 2; OS: overall survival

**Table 3:** Univariate and multivariate analyses of ACP2 with regard to OS in stage III colorectal cancer

Variables	Comparison	HR^a^	95% CI^b^	*P*	HR^a^	95% CI^b^	*P*
Cox Univariate analysis (OS)				Cox Multivariate analysis (OS)			
Tumor	T12 vs. T34	1.042	0.413–2.630	0.930	0.735	0.278–1.945	0.535
Emboli	Yes;No	1.500	0.766–2.937	0.236	1.496	0.746–2.999	0.257
Perineural	Yes;No	1.498	0.843–2.663	0.169	1.613	0.886–2.936	0.118
Chemotherapy	Yes;No	0.519	0.265–1.017	0.056	0.456	0.227–0.917	0.027
ACP2	Low;High	1.054	0.600–1.851	0.855	1.073	0.606–1.900	0.810

^a^ Hazard ratio (HR) estimated from Cox proportional hazard regression model.

^b^ Confidence interval of the estimated HR.

ACP2: Acid phosphatase 2; OS: overall survival

### Significance of ACP2 expression in response to adjuvant chemotherapy

**Figure 2:**
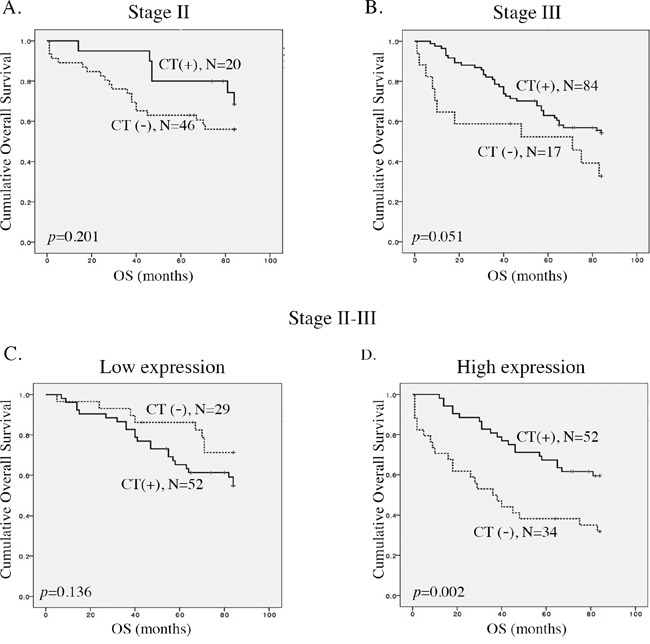
Correlation between ACP2 expression and the benefit of adjuvant chemotherapy **A** and **B**. Kaplan–Meier plots of overall survival for stage II and stage III colorectal cancer (CRC) patients with or without adjuvant chemotherapy (CT). **C** and **D**. Kaplan–Meier plots of overall survival for low and high levels of expression of ACP2 in stage II and III CRC patients.

Chemotherapy regimens in CRC patients are primarily 5-FU based. Regardless of ACP2 expression status, we found that patients who had received adjuvant chemotherapy tended to have better survival, but without significance for stage III CRC (*p* = 0.051; Figure 2A, 2B). We then analyzed ACP2 expression and its relation to chemotherapy. Surprisingly, stage II and III CRC patients with high expression of ACP2 showed significantly better seven-year survival in response to chemotherapy compared with those with low expression of ACP2 in stage II and III CRC (*P* = 0.002 *versus p* = 0.136; Figure 2C and 2D).

**Figure 3:**
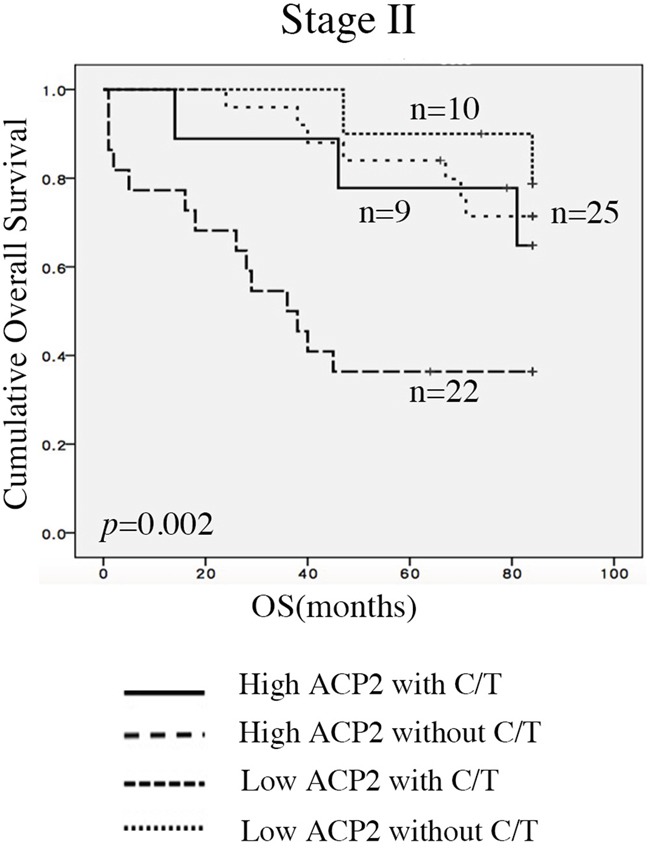
Associations between ACP2 expression and adjuvant chemotherapy Expression of ACP2 levels with or without adjuvant chemotherapy (CT) in stage II colorectal cancer (CRC) patients.

A combinatorial analysis of ACP2 expression and adjuvant chemotherapy was conducted separately for stage II and III CRC. In stage II CRC, high ACP2 expression without chemotherapy had the worst survival as compared with the other three groups (*p* = 0.002; Figure 3). In the stage III CRC, a high ACP2 expression without chemotherapy (n = 10) also had the worst survival (*P* = 0.044; Supplementary Figure 2), confirming that high ACP2 expression was an unfavorable prognostic factor. Furthermore, we found that CRC patients (n = 4) with low ACP2 expression and without chemotherapy showed the best survival. These results indicate that patients with high ACP2 expression gained more benefit from chemotherapy than those with low ACP2 expression. These results demonstrate the potential of ACP2 expression to be used as a prognostic marker to identify CRC patients who can benefit from adjuvant chemotherapy.

### Knockdown of ACP2 expression may enhance chemoresistance

**Figure 4:**
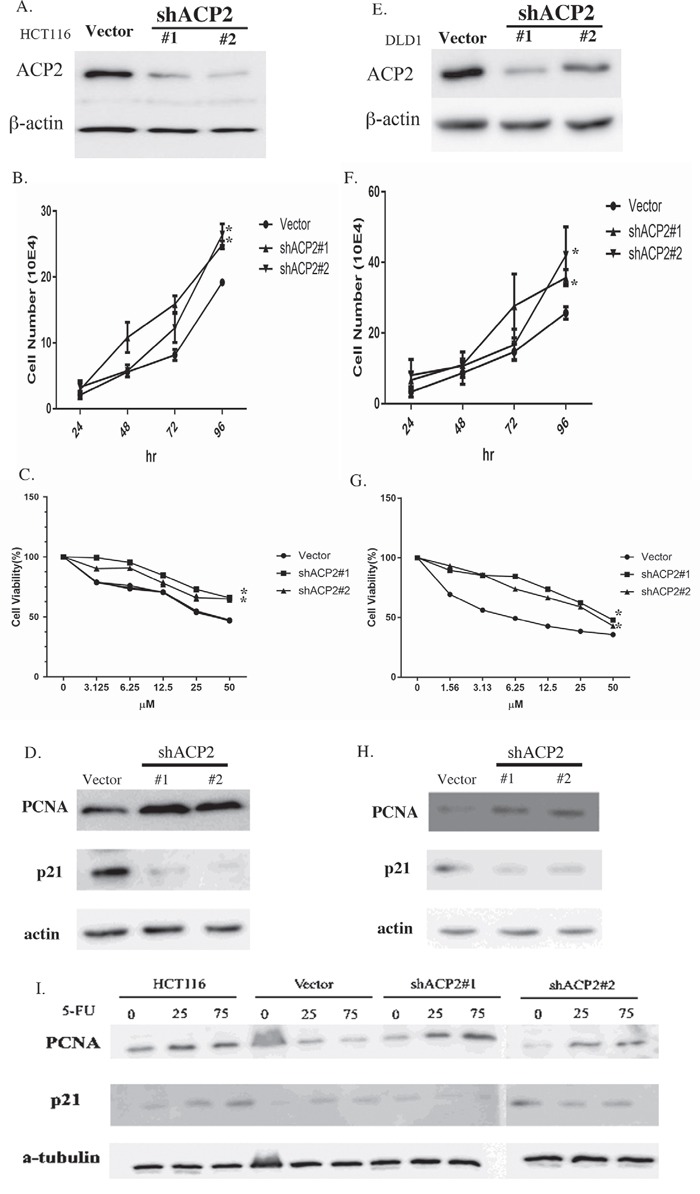
Knockdown of ACP2 expression enhances chemoresistance ACP2 expression was knocked down in human colorectal cancer cell lines HCT116 **A–D**. and DLD1 **E–H**. A/E. Western blot results for ACP2 knockdown. B/F. Cell numbers per 24 h. C/G. MTT assay for each concentration of 5-fluorouracil (5-FU). D/H. Expression of proliferating cell nuclear antigen (PCNA) and p21 detected by Western blot assay. **I**. A Western blot assay showing the effect of ACP2 and 5-FU treatment on the level of PCNA and p21 (in the HCT116 cell line).

As shown in Figure 2D, ACP2 high expression of patients to adjuvant 5-FU have more best prognosis. Therefore, we thought that high expression of ACP2 in CRC patients was associated with increased sensitivity to 5-FU chemotherapy. To examine whether this was because the expression of ACP2 induced chemosensitivity, we used the shACP2 lentivirus to knock down ACP2 expression in human CRC cell lines HCT-116 and DLD1 after we examined the expression level of ACP2 in six colon cancer cell lines (Supplementary Figure 1). The level of ACP2 protein expression in cells was determined by Western blotting analysis (Figure 4A, 4E). To understand whether the expression of shACP2 affected cell growth, we performed a cell proliferation assay. As expected, cell counting revealed that downregulation of ACP2 expression induced the proliferation of HCT116 and DLD1 cells (Figure 4B, 4F). To confirm that ACP2-knockdown cells were more resistant to 5-FU treatment compared with vector control by MTT assay (Figure 4C, 4G). We have calculated the IC_50_ values (Supplementary Table 2). We found that the downregulation of ACP2 expression could significantly induce the HCT-116 and DLD1 cells to survive 5-FU treatment compared with control. Furthermore, we investigated the expression of PCNA and p21 in shACP2 cells compared with vector control. Our results showed knockdown of ACP2 expression increased PCNA expression and reduced p21 expression in both HCT116 and DLD1 cells (Figure 4D, 4H). In addition, we investigated the effect of 5-FU on the knockdown of ACP2 expression in HCT116 cells treated with different concentrations of 5-FU (0, 50, and 75 μM) for 48 h. The results showed that, compared with vector control, the expression of PCNA increased with the concentration of 5-FU treatment (Figure 4I).

**Figure 5:**
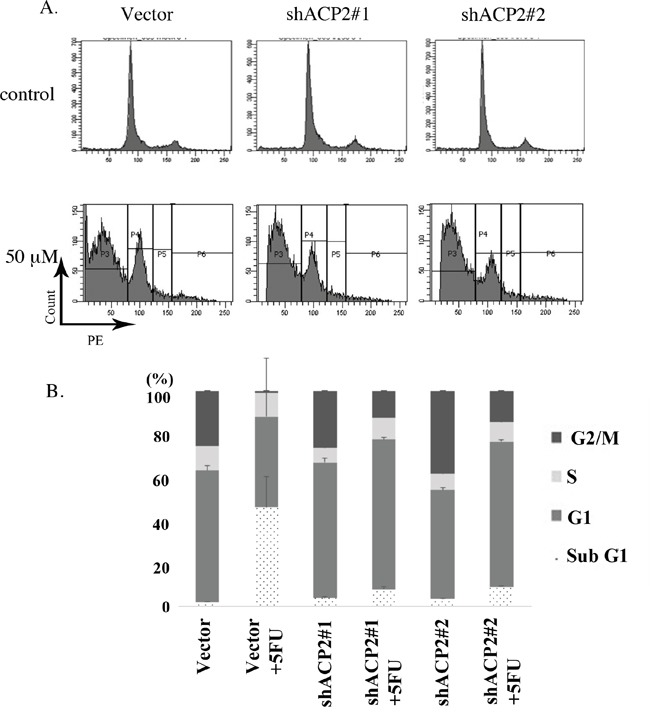
Knockdown of ACP2 expression inhibited the subG1 population **A**. Knockdown of ACP2 cells caused the inhibition of cells in the subG1 phase compared with vector control by flow cytometry. **B**. Data representing the distributions of cell types as percentages.

In examining whether knockdown of ACP2 expression could increase resistance to 5-FU, further analysis of the cell cycle showed that, under treatment with 5-FU, the control cells had greater subG1 phase cell accumulation (43.50%) compared with that of shACP2#1 (7.63%), shACP2#2 (8.87%). The ACP2-knockdown cells significantly inhibited cells in the subG1 phase, with a concomitant decrease of cells in the G2-M phase compared with vector control cells (Figure 5A, 5B).

Taken together, these results indicated that the knockdown of ACP2 markedly increased cell proliferation, inhibited cell apoptosis, and resistant to chemotherapy in CRC cells. These data were consistent with our clinical results.

### Knockdown of ACP2 expression with adjuvant 5-FU enhanced chemoresistance and tumor migration/invasion

**Figure 6:**
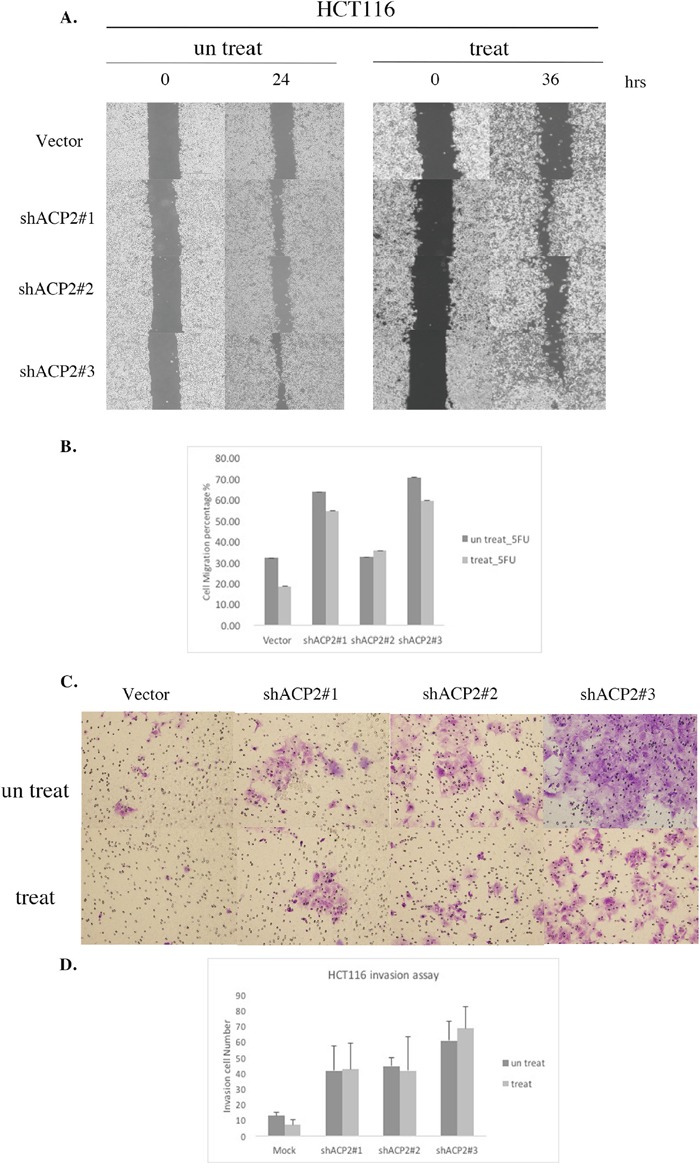
Knockdown of ACP2 expression induced cell migration and invasion activity in HCT116 cells HCT116 cell infection with vector and ACP2 plasmids was used for migration A. and quantitative B. invasion assay C. and quantitative D. Five fields of cells in the lower side were counted. Data are presented as means ± SEM of three independent experiments.

In 2015, Holle et al. reported that cancer cells or tumors could bring resistance through creating highly organized extracellular matrix structures that inhibit drug penetration [13]. For this reason, we future examined to cell migration and invasion abilities were detected in ACP2 knockdown HCT116 cells and DLD1 cells with or without 5-FU treatment in a transwell assay. The results suggested that knockdown of ACP2 enhanced migration and invasion activity both with and without treatment with 5-FU (Figure 6). These findings indicated the potential for ACP2 expression to act as a predictor of the level of success of adjuvant chemotherapy in colon cancer.

## DISCUSSION

Currently, adjuvant chemotherapy affects prognosis and survival in stage III and high-risk stage II patients of CRC. By now, the first steps in the risk assessment for CRC are pathologic tumor stage and mismatch repair (MMR) status. In general, CRC patients of stage II classified a low risk condition normally are not recommended for chemotherapy. The GeneFxColon, OncoDefender-CRC and ColoGuideEx are well-developed to identify special patients with stage II CRC, which mostly equal to a higher risk of relapse and recommended for chemotherapy [14]. However, the abovementioned analyses have still not been applicable in a clinical setting for the risk of reliable prospective, independent validation [15] and issue of cost [16]. High microsatellite instability (MSI-H), the most reliable analysis, is a good prognostic factor for stage II CRC patients, but ineffective in benefit prediction for adjuvant therapy. Only 15% of CRC patients were MSI-H positive in a previous study [17] and 10% in Taiwan [18, 19]. In this study, we analyzed the relationship of ACP2 expression with chemotherapy requirement and the outcome of the patients analyzed. The results revealed that ACP2 was an unfavorable prognostic marker for stage II CRC. Nevertheless, concomitant chemotherapy targeting ACP2 had significantly better survival in stage II CRCs. These findings indicate that ACP2 was not only a prognostic factor but also a novel biomarker for the prediction of chemotherapeutic success for high-risk patients with stage II CRC.

It is well known that T stage stands for a risky indicator in stage II CRC patients [20], which was consistent with the data shown in Table 2. Further analysis using multivariate Cox proportional hazards regression suggested that T stage and ACP2 expression were independent prognostic markers for the overall survival in stage II CRC. Therefore, commination of T stage and ACP2 expression provides effective diagnostic criteria of CRC patients with chemotherapy. In contrast to a similar assay for stage III CRC patients, they did not show a significant relationship (Table 3). ACP2 expression serving as a potential predictor to treatment response is more reliable for stage II than stage III CRC.

Melquist et al. has shown the block of microtubule-associated protein tau (MAPT) H1 haplotype associated with parkinsonism; the ACP2 gene is involved in this scenario. In this connection, lysosomal dysfunction has been incriminated in aging and neurodegeneration [20]. As aforementioned, ACP2 plays an important role in developmental biology. It is well known that development-related genes promoted carcinogenesis, in which cells featured abnormal differentiation or proliferation by itself [21]. Cell proliferation in irregular conditions increased the incident rate of tumor formation by treatment with 2-acetylaminofluorene, a potential human carcinogen [22]. The downregulation of ACP2 expression increased PCNA expression and decreased p21 expression, resulting in the proliferation of HCT116 and DLD1 cells and more resistance to 5-FU treatment (Figure 4). The present findings bolster the high expression of ACP2 in tumor cells, which may link to cell fate.

ACP2 is located in lysosomes and involved in the formation of autolysosomes in autophagy mechanics. Hiroshi et al., had shown that the knockdown of Beclin1, a well-known autophagy gene, induced cell proliferation in human ovarian clear cell carcinomas [23], and provoked chemoresistance [24]. The resulted chemoresistance in gastric cancer was also evidenced in the suppression of ING5, which stimulated tumor cell migration and invasion in concurrence with the inhibition of autophagy [25]. The autophagy invalidation was well known in the knockdown of another autophagy gene, Apg7, which consequently reduced apoptotic cells [26]. The suppression of autophagy could hence trigger an enhanced chemoresistance. Additionally, recent studies have shown that enhance of drug-resistance resulted in cell migration and invasion ability [27–29]. A study by Liu et al. demonstrated that HepG2 cell line resistant to sorafenib showed enhanced cell migration and invasion capability and decreased cell apoptosis. Chi et al. reported that drug resistance in cancer could promote cell growth, metastasis, and invasion. These results supported the hypothesis that chemoresistance may promote cellular migration and invasion which has also been shown in our results”. In this study, the knockdown of ACP2, leading to 5-FU resistance, decreased apoptotic cells (Figure 4), and Becline 1 and Apg7 levels (Supplementary Figure 3). In this connection, ACP2 may be involved in chemoresistance through autophagy.

In conclusion, the present study demonstrated that the extent of ACP2 expression in patients might be a novel prognostic and therapeutic predictor marker for stage II CRC. Adjuvant chemotherapy is highly recommended for Stage II CRC patients with high ACP2 expression.

## MATERIALS AND METHODS

### ACP2 survival analysis using online databases

RNA microarray analysis results of 111 patients with CRC were collected from the GSE database (GSE 17536) to evaluate the correlation between ACP2 expression and patient outcomes. The average expression level of all ACP2 probes with quantile normalization were used to represent the RNA expression level of ACP2. The samples were splited into two risk groups of the same size according to the prognostic index estimated from beta coefficients multiplied by gene expression values.

### Specimens

Specimens from a total of 167 patients with stage II and III CRC who received colonic resection were included in this study. The patients were followed up for 84 months. Formalin-fixed paraffin-embedded surgically removed CRC tumor tissues were retrieved from Wan Fang Hospital following the approval of the Institutional Review Board (WFH-IRB-99049). Pathological diagnoses of each case was confirmed by pathologists (CYS, MH, and CLC) in accordance with the WHO classification. The adequate formalin-fixed paraffin-embedded surgical specimen tissues of each case were carefully selected and a 1-mm-diameter tissue core of each tumor was taken to construct the tissue microarray.

### Immunohistochemistry staining and analysis

Immunohistochemical (IHC) staining was performed by automated immunostainer (Ventana Discovery XT autostainer, Ventana Medical Systems Inc.) on 5-μm-thick tissue sections cut from the tissue microarray. Briefly, after dewaxed thoroughly xylene and ethanol, antigen retrieval was performed using a citrate buffer at pH 6.5 for 8 min. The sections were then reacted with polyclonal rabbit ACP2 antibody (1:400; GeneTex, Irvine, CA, USA). The sections were counterstained with hematoxylin after developed with diaminobenezidine (DAB). The IHC results were separately scored by two pathologists (CLC and CYS), scoring ACP2 expression according to a four-tiered approach: negative (0), weak (1+), moderate (2+), and strong (3+). Low ACP2 expression was defined as negative (0) or weak expression (1+), and high ACP2 expression as moderate (2+) or strong expression (3+).

### Cell cultures

The human CRC cell line HCT-116 was purchased from ATCC (CCL-247) and was maintained in RPMI 1640 Medium with 1% penicillin/streptomycin, 10% (v/v) FBS at 37 °C, and 5% CO_2_.

### Cell proliferation

5×10^4^ cells were seeded in a 24-well plate and were cultured in complete RPMI 1640 or DMEM containing 10% FBS for cell proliferation assay. The cell numbers were counted every 24 h using Countess™ (Invitrogen, Carlsbad, CA, USA).

### Lentivirus-based shRNA production and infection

Lentivirus-based production and infection followed a previously published protocol [30]. Briefly, the lentiviral shRNA constructs were purchased from Thermo Fisher Scientific (Pittsburgh, PA, USA). Lentiviruses were produced by calcium phosphate transfection method (Invitrogen) and created by co-transfection of shRNA-expressing plasmids, envelope plasmids (pMD.G) and packaging plasmids (pCMV-dR8.91) in 293T cells. The transfected 293T cells were incubated in the culture medium for 18 h; the supernatant was then removed and refreshed. The supernatants containing the shRNA viruses were harvested and tittered at 72 h post-transfection. CRC cells were infected with the lentiviruses in the presence of Polybrene (8 μg/mL), and were proceeded enrich infected cell by puromycin selection (2 μg/mL).

### Western blot analysis

Cells were lysed at 4 °C in RIPA buffer supplemented with protease and phosphatase inhibitors. Equal amounts of proteins were separated by using sodium dodecyl sulfate polyacrylamide gels, and then transferred to a PVDF membrane (Millipore, Bedford, MA, USA). After blocking with 5% non-fat milk, the membrane was incubated with specific antibodies overnight at 4 °C and then incubated with horseradish peroxidase (HRP) conjugated secondary antibody for 1 h. The blots were visualized by using an ECL-Plus detection kit (PerkinElmer Life Sciences, Boston, MA, USA) [31].

### Cell viability assay

Cell viability was determined by using the standard MTT assay. Briefly, cells (5 × 10^3^ cells/well) were grown on a 96-well plate supplemented with culture medium. After overnight incubation, the cells were treated with 5-FU for 72 h. At the end of treatment, 20 μL of MTT (5 mg/mL) was added to the culture medium and incubation continued at 37 °C for additional 4 h. The absorbance was measured with a spectrophotometer at 560 nm. A blank with DMSO alone was measured as a background control [31].

### Cell cycle analysis

To assess the cell cycle distribution under drug treatment, cells were seeded at a density of 2 × 10^5^ cells/well in six-well plates in RPMI 1640 for 24 h. Cells were then incubated in 2 mL of complete RPMI 1640 with 50 μM of 5-FU for 48 h. After treatment, cells were washed once with phosphate-buffered saline and then harvested by trypsinization. For flow cytometry, cells were briefly incubated with 10 μg/mL RNase A at 37 °C for 15 min and then stained with 5 μg/mL Hochest 33342 and 20 μg/mL PI for 10 min at room temperature away from light. Ten thousand cells per sample were analyzed using a BD FACSCanto II Flow Cytometer (Becton, Dickinson and Co., San Jose, CA) [32].

### Migration assay

The physical wounding of cell cultures was used to assess cell migration (ibidi, see http://www.ibidi.de). Cells were seeded at 3 × 10^5^ cells per chamber and allowed to attach overnight with or without 5-FU treatment. The following day, culture inserts were removed and light microscopy images acquired (three per condition). Cells were cultured during the migration assay and incubator in normal conditions, and images were acquired 24 h and 36 h later. Images were analyzed using ImageJ software.

### Invasion assay

8-mm polycarbonate filters were coated with Matrigel on the lower side, with 3 × 10^5^ cells loaded on the upper chamber. After 6 h, half the cells were treated with 5-FU. After 24 h or 42 h, the membranes were fixed and stained with 0.2% crystal violet/20% methanol. Quantification was performed by counting the stained cells. The invaded cells were counted under light microscope (200 fold, five random fields from each well). All experiments were performed in triplicate.

### Statistical analysis

Results are presented as mean ± SD. Two-tail unpaired Student *t* tests were used for all pairwise comparisons. Survival curves were analyzed by the log-rank Kaplan–Meier method. Cox proportional hazards regression was applied to test the prognostic factors in univariate and multivariate models. *P* values less than 0.05 were considered to be significant.

## SUPPLEMENTARY MATERIALS FIGURES AND TABLES



## References

[1] Ferlay J, Steliarova-Foucher E, Lortet-Tieulent J, Rosso S, Coebergh JW, Comber H, Forman D, Bray F (2013). Cancer incidence and mortality patterns in Europe: estimates for 40 countries in 2012. Eur J Cancer.

[2] Griffin MR, Bergstralh EJ, Coffey RJ, Beart RW, Melton LJ (1987). Predictors of survival after curative resection of carcinoma of the colon and rectum. Cancer.

[3] Kuebler JP, Wieand HS, O’Connell MJ, Smith RE, Colangelo LH, Yothers G, Petrelli NJ, Findlay MP, Seay TE, Atkins JN, Zapas JL, Goodwin JW, Fehrenbacher L (2007). Oxaliplatin combined with weekly bolus fluorouracil and leucovorin as surgical adjuvant chemotherapy for stage II and III colon cancer: results from NSABP C-07. J Clin Oncol.

[4] Kelder W, Hospers GA, Plukker JT (2006). Effects of 5-fluorouracil adjuvant treatment of colon cancer. Expert Rev Anticancer Ther.

[5] Isacoff WH, Borud K (1997). Chemotherapy for the treatment of patients with metastatic colorectal cancer: an overview. World J Surg.

[6] Hamburg MA, Collins FS (2010). The path to personalized medicine. N Engl J Med.

[7] Waheed A, Gottschalk S, Hille A, Krentler C, Pohlmann R, Braulke T, Hauser H, Geuze H, von Figura K (1988). Human lysosomal acid phosphatase is transported as a transmembrane protein to lysosomes in transfected baby hamster kidney cells. EMBO J.

[8] Saftig P, Hartmann D, Lullmann-Rauch R, Wolff J, Evers M, Koster A, Hetman M, von Figura K, Peters C (1997). Mice deficient in lysosomal acid phosphatase develop lysosomal storage in the kidney and central nervous system. J Biol Chem.

[9] Nadler HL, Egan TJ (1970). Deficiency of lysosomal acid phosphatase. A new familial metabolic disorder. N Engl J Med.

[10] Mannan AU, Roussa E, Kraus C, Rickmann M, Maenner J, Nayernia K, Krieglstein K, Reis A, Engel W (2004). Mutation in the gene encoding lysosomal acid phosphatase (Acp2) causes cerebellum and skin malformation in mouse. Neurogenetics.

[11] Somoza-Martin JM, Garcia-Garcia A, Barros-Angueira F, Otero-Rey E, Torres-Espanol M, Gandara-Vila P, Reboiras-Lopez MD, Blanco-Carrion A, Gandara-Rey JM (2005). Gene expression profile in oral squamous cell carcinoma: a pilot study. J Oral Maxillofac Surg.

[12] Smith JJ, Deane NG, Wu F, Merchant NB, Zhang B, Jiang A, Lu P, Johnson JC, Schmidt C, Bailey CE, Eschrich S, Kis C, Levy S (2010). Experimentally derived metastasis gene expression profile predicts recurrence and death in patients with colon cancer. Gastroenterology.

[13] Holle AW, Young JL, In Spatz JP (2016). vitro cancer cell-ECM interactions inform in vivo cancer treatment. Adv Drug Deliv Rev.

[14] Kopetz S, Tabernero J, Rosenberg R, Jiang ZQ, Moreno V, Bachleitner-Hofmann T, Lanza G, Stork-Sloots L, Maru D, Simon I, Capella G, Salazar R (2015). Genomic classifier ColoPrint predicts recurrence in stage II colorectal cancer patients more accurately than clinical factors. Oncologist.

[15] Giraldez MD, Lozano JJ, Cuatrecasas M, Alonso-Espinaco V, Maurel J, Marmol M, Horndler C, Ortego J, Alonso V, Escudero P, Ramirez G, Petry C, Lasalvia L (2013). Gene-expression signature of tumor recurrence in patients with stage II and III colon cancer treated with 5’fluoruracil-based adjuvant chemotherapy. Int J Cancer.

[16] You YN, Rustin RB, Sullivan JD (2015). Oncotype DX((R)) colon cancer assay for prediction of recurrence risk in patients with stage II and III colon cancer: A review of the evidence. Surg Oncol.

[17] Kumar A, Kennecke HF, Renouf DJ, Lim HJ, Gill S, Woods R, Speers C, Cheung WY (2015). Adjuvant chemotherapy use and outcomes of patients with high-risk versus low-risk stage II colon cancer. Cancer.

[18] Lin CC, Lai YL, Lin TC, Chen WS, Jiang JK, Yang SH, Wang HS, Lan YT, Liang WY, Hsu HM, Lin JK, Chang SC (2012). Clinicopathologic features and prognostic analysis of MSI-high colon cancer. Int J Colorectal Dis.

[19] Fang WL, Chang SC, Lan YT, Huang KH, Chen JH, Lo SS, Hsieh MC, Li AF, Wu CW, Chiou SH (2012). Microsatellite instability is associated with a better prognosis for gastric cancer patients after curative surgery. World J Surg.

[20] Melquist S, Craig DW, Huentelman MJ, Crook R, Pearson JV, Baker M, Zismann VL, Gass J, Adamson J, Szelinger S, Corneveaux J, Cannon A, Coon KD (2007). Identification of a novel risk locus for progressive supranuclear palsy by a pooled genomewide scan of 500,288 single-nucleotide polymorphisms. Am J Hum Genet.

[21] Thu KL, Becker-Santos DD, Radulovich N, Pikor LA, Lam WL, Tsao MS (2014). SOX15 and other SOX family members are important mediators of tumorigenesis in multiple cancer types. Oncoscience.

[22] Cohen SM, Ellwein LB (1990). Cell proliferation in carcinogenesis. Science.

[23] Katagiri H, Nakayama K, Razia S, Nakamura K, Sato E, Ishibashi T, Ishikawa M, Iida K, Ishikawa N, Otsuki Y, Nakayama S, Kyo S (2015). Loss of autophagy-related protein Beclin 1 may define poor prognosis in ovarian clear cell carcinomas. Int J Oncol.

[24] Ying H, Qu D, Liu C, Ying T, Lv J, Jin S, Xu H (2015). Chemoresistance is associated with Beclin-1 and PTEN expression in epithelial ovarian cancers. Oncol Lett.

[25] Gou WF, Shen DF, Yang XF, Zhao S, Liu YP, Sun HZ, Su RJ, Luo JS, Zheng HC (2015). ING5 suppresses proliferation, apoptosis, migration and invasion, and induces autophagy and differentiation of gastric cancer cells: a good marker for carcinogenesis and subsequent progression. Oncotarget.

[26] Law BY, Chan WK, Xu SW, Wang JR, Bai LP, Liu L, Wong VK (2014). Natural small-molecule enhancers of autophagy induce autophagic cell death in apoptosis-defective cells. Sci Rep.

[27] Liu K, Liu S, Zhang W, Jia B, Tan L, Jin Z, Liu Y (2015). miR-494 promotes cell proliferation, migration and invasion, and increased sorafenib resistance in hepatocellular carcinoma by targeting PTEN. Oncol Rep.

[28] Li H, Zhang P, Sun X, Sun Y, Shi C, Liu H, Liu X (2015). MicroRNA-181a regulates epithelial-mesenchymal transition by targeting PTEN in drug-resistant lung adenocarcinoma cells. Int J Oncol.

[29] Chi JY, Hsiao YW, Li CF, Lo YC, Lin ZY, Hong JY, Liu YM, Han X, Wang SM, Chen BK, Tsai KK, Wang JM (2015). Targeting chemotherapy-induced PTX3 in tumor stroma to prevent the progression of drug-resistant cancers. Oncotarget.

[30] Su CY, Lin TC, Lin YF, Chen MH, Lee CH, Wang HY, Lee YC, Liu YP, Chen CL, Hsiao M (2015). DDX3 as a strongest prognosis marker and its downregulation promotes metastasis in colorectal cancer. Oncotarget.

[31] Lin TC, Liu YP, Chan YC, Su CY, Lin YF, Hsu SL, Yang CS, Hsiao M (2015). Ghrelin promotes renal cell carcinoma metastasis via Snail activation and is associated with poor prognosis. J Pathol.

[32] Lee YC, Lee CH, Tsai HP, An HW, Lee CM, Wu JC, Chen CS, Huang SH, Hwang J, Cheng KT, Leiw PL, Chen CL, Lin CM (2015). Targeting of Topoisomerase I for Prognoses and Therapeutics of Camptothecin-Resistant Ovarian Cancer. PLoS One.

