# Supplementary material for: Lysosomal acid phosphatase 2 is an unfavorable prognostic factor but is associated with better survival in stage II colorectal cancer patients receiving chemotherapy
Source: Oncotarget. 2017 Jan 6;8(7):12120–32. doi: 10.18632/oncotarget.14552 (PMC5355330; doi:10.18632/oncotarget.14552)
Supplement: Supplementary file 1 [file oncotarget-08-12120-s001.pdf]

Lysosomal acid phosphatase 2 is an unfavorable prognostic factor but is associated with better survival in stage II colorectal cancer patients receiving chemotherapy

SUPPLEMENTARY FIGURES AND TABLES

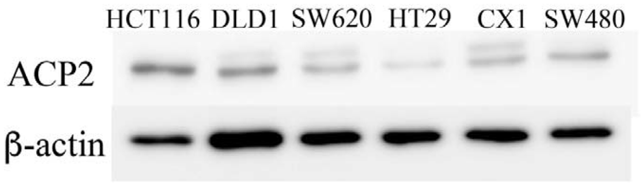

Supplementary Figure 1: Protein expression of Human colorectal cancer cell lines.

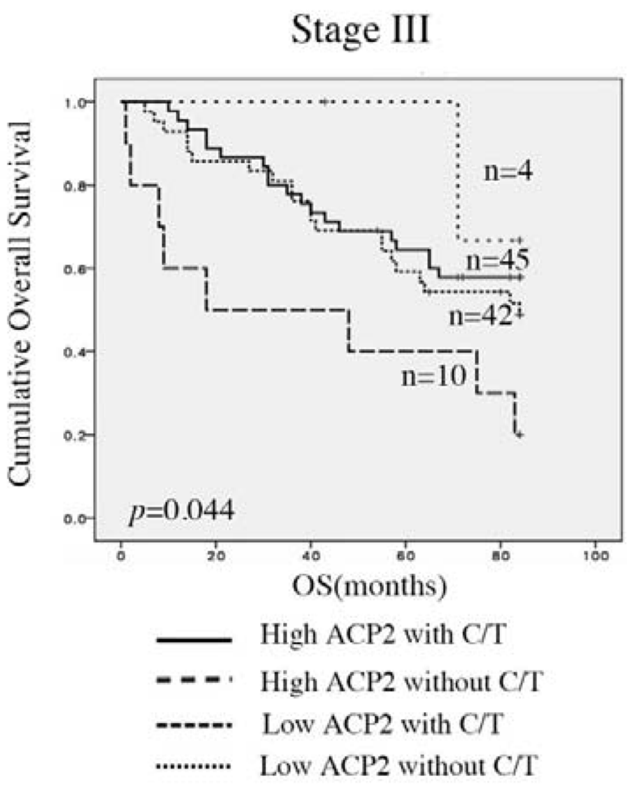

Supplementary Figure 2: Expression of ACP2 levels with or without adjuvant CT in stage III CRC patients.

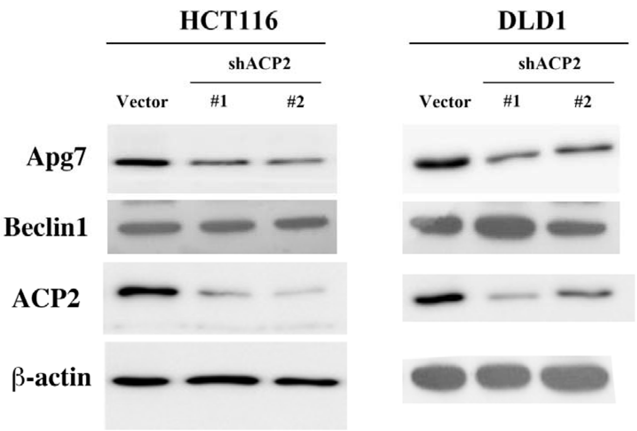

Supplementary Figure 3: ACP2 knockdown in HCT-116 and DLD1 colon cancer cells suppresses autophagy marker Apg7 and not Beclin1.

Supplementary Table 1: Association of ACP2 expression with clinicopathological features in stage II and III of colorectal cancer

Clinical factor	ACP2 low n (%)	ACP2 high n (%)	*P*
T			0.447
1	0 (0)	1 (1.2)	
2	4 (4.9)	5 (5.8)	
3	72 (88.9)	70 (81.4)	
4	5 (6.2)	10 (11.6)	
N			0.35
0	35 (43.2)	31 (36)	
1	46 (56.8)	55 (64)	
Emboli			0.630
NO	35 (43.2)	34 (39.5)	
YES	46 (56.8)	52 (60.5)	
Perineural			0.562
NO	61 (75.3)	68 (79.1)	
YES	20 (24.7)	18 (20.9)	
Chemotherapy			0.619
NO	29 (35.8)	34 (39.5)	
YES	52 (64.2)	52 (60.5)	
Recurrence			0.689
NO	60 (74.1)	66 (76.7)	
YES	21 (25.9)	20 (23.3)	

Supplementary Table 2: The IC50 values for 5FU treatment in downregulated of ACP2 in HCT116 and DLD1 cells

IC50(μM)	Vector	shACP2#1	shACP2#2
HCT116	38.96	<50	<50
DLD1	5.91	45.51	38.51
